# A 26-Year-Old Male with Mesothelioma Due to Asbestos Exposure

**DOI:** 10.1155/2011/951732

**Published:** 2011-07-12

**Authors:** P. Zarogoulidis, M. Orfanidis, T. C. Constadinidis, E. Eleutheriadou, T. Kontakiotis, T. Kerenidi, L. Sakkas, N. Courcoutsakis, K. Zarogoulidis

**Affiliations:** ^1^Oncology Unit, Pulmonary Department, “G. Papanikolaou” General Hospital, Aristotle University of Thessaloniki, Exohi, 57010 Thessaloniki, Greece; ^2^Laboratory of Hygiene and Environmental Protection and Regional Laboratory of Public Health, Medical School, Democritus University of Thrace, Eastern Macedonia-Thrace, 68100 Alexandroupolis, Greece; ^3^Pulmonary Clinic, University of Thessaly, 38221 Larisa, Greece; ^4^Pathology Department, “G. Papanikolaou” General Hospital, 57010 Thessaloniki, Greece; ^5^Department of Radiology, Democritus University of Thrace, 68100 Alexandroupolis, Greece

## Abstract

Mesothelioma is a malignancy with poor prognosis, with an average 5-year survival rate being less than 9%. This type of cancer is almost exclusively caused by exposure to asbestos. A long exposure can cause mesothelioma and so can short ones, as each exposure is cumulative. We report a case of a 26-year-old male who was exposed to asbestos during his primary school years from the age of 6 to 12. Although the tumor mainly affects older men who in their youth were occupationally exposed to asbestos, malignant mesothelioma can also occur in young adults. A medical history was carefully taken and asbestos exposure was immediately mentioned by the patient. We conducted biopsy on the right supraclavicular lymph node. The patient was not a candidate for surgery, and chemotherapy treatment was initiated. While patient's chemotherapy is still ongoing, no other similar cases of students or teachers have been traced up to date from his school. The school building was demolished in January 2009.

## 1. Introduction

Asbestos defines a group of naturally occurring mineral silicates, which are used for various commercial applications including fireproofing and insulation. Asbestos readily breaks into small dust-like fibers, which are easily inhaled, resulting in a variety of diseases of the respiratory system including asbestosis, lung cancer, and malignant pleural mesothelioma (MPM) [1, 2]. To date, asbestos has largely been banned from work and home environments, but many countries still produce and export asbestos under strict regulations and at reduced levels [3, 4]. Asbestos-related lung diseases will remain a major health concern, mainly due to the long latency between exposure and disease development [4, 5]. Once asbestos exposure has occurred, there is no secondary prevention of chest diseases or prophylactic treatment available. There is a direct causal relation between the degree of asbestos exposure and MPM [6, 7]. While the risk of lung cancer slowly declines after smoking cessation, the risk of MPM progressively increases with time [8]. Pleural plaques and MPM share the same etiology but have a different latency period (10–20 years for plaques, 20–40 years for MPM), and even though plaques are not regarded as a risk factor of MPM, the presence of pleural plaques provides a risk indicator and thus a window of opportunity for MPM diagnosis [9–14]. The diagnosis is further complicated by the wide variety of histological appearances that mesotheliomas may assume and the large number of entities that can simulate this appearance. Therefore, a diagnosis of mesothelioma should be made only after cautious consideration of the available information.

## 2. Case Report

**Figure 1:**
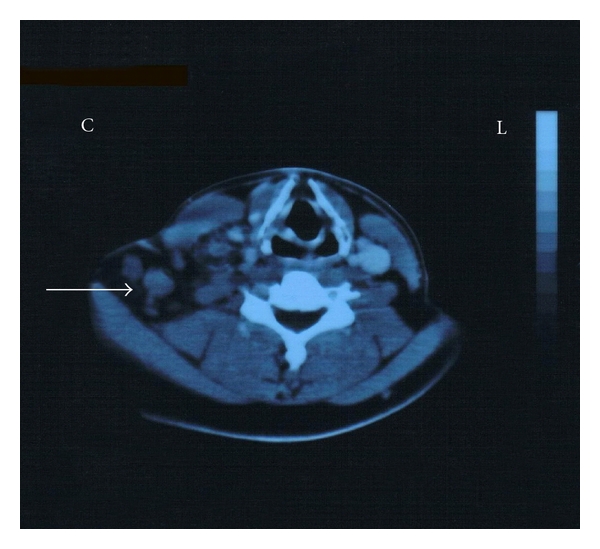
Enlarged supraclavicular node.

**Figure 2:**
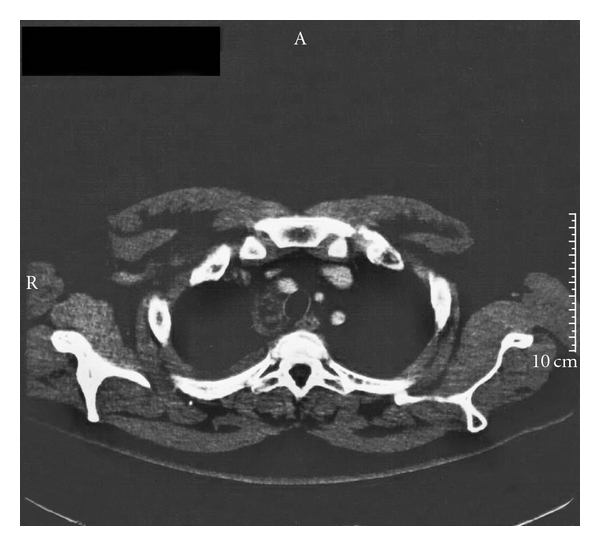
Axial enhanced CT-image of paratracheal right-sided mass and pleural effusion.

**Figure 3:**
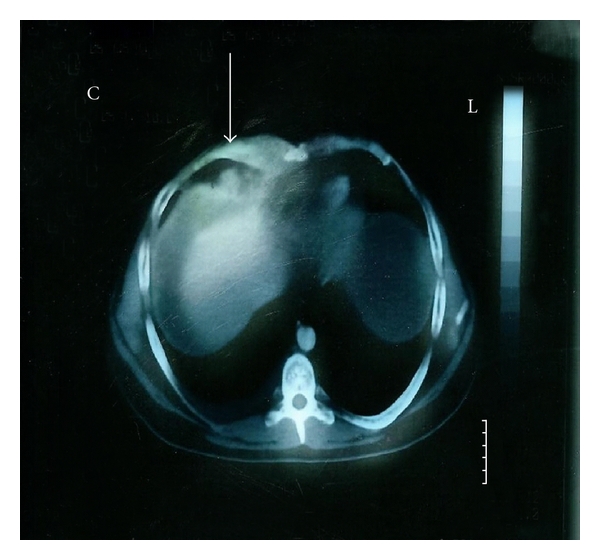
Axial enhanced CT-image of the lower part of the chest (mediastinal window) shows small pleural effusion and an epiphrenic node at the right. Ascites and thickness of the right crus are also noticed.

**Figure 4:**
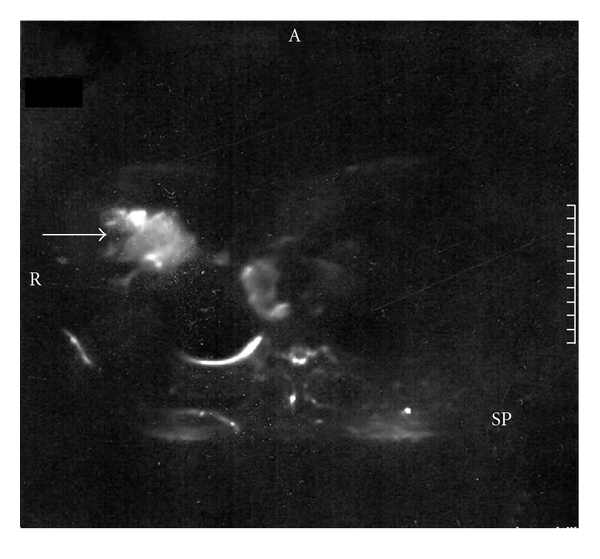
Axial stir (short-tau-invesion-recovery) MR image of the upper thorax demonstrates a lesion located in the right part of the anterior thoracic wall and shows increased inhomogeneous signal intensity. The lesion has irregular contour, involving the major pectoralis muscle. Inside the lesion, a node is noticed.

A-26-year-old male attended the emergency department with intense pain, of a right-sided supraclavicular lump, with signs of visible inflammation. The history of the presented lymph node revealed that the swollenness was initially presented three months ago. In spite of the gradual enlargement, the patient did not seek any medical advice or treatment. On chest palpation, there was edema, redness, and lymphatic obstruction of the right tracheal position, pectoralis, shoulder, and humerus. There was no abnormality of chest expansion and symptoms of fever, night sweats, and infection. The patient complained of fatigue that gradually escalated during the past 3 weeks, but without any weight loss. On lung percussion, the right side was equal to the left and resonant. On lung auscultation, there were vesicular breath sounds and there was no reduced air entry, no crackles, no wheeze, and no crepitating or rub. Clinical examination of the abdomen revealed ascites, and abdominal U/S confirmed peritoneum effusion (exudate). There were no other pathologic findings on cardiovascular and neurological examination. Past medical history did not reveal any previous medical problems or operations. The patient was on no regular medication. Social history excluded smoking, illegal drug, and alcohol abuse. The chest X-ray showed no pathology. On further questioning, the patient mentioned that his school building was constructed with asbestos-containing materials and that it was recently demolished by the local prefecture authorities. This exposure was the only asbestos exposure he had during his life. During hospitalization, he developed an acute right-sided chest pain. His ECG was normal; however, the CT scan of the trachea demonstrated an enlarged supraclavicular node (Figure 1), and on the CT scan of the thorax (Figure 2), nodes between the vessels of the upper mediastinum were demonstrated. In addition, small pleural effusion and an epiphrenic node on the right were observed (Figure 3). Further evaluation of the patient was done to evaluate the local extension of the lesion beginning from the right supraclavicular node to the right pectoralis with MRI. The MRI of the upper thorax revealed that the lesion had irregular contour, involving the major pectoralis muscle (Figure 4). At that time, the possibility of mesothelioma was considered, as there were no other clinical or laboratory findings supporting any specific diagnoses. Diffuse malignant mesothelioma arises from the mesothelium. It can occur in any of the body cavities covered by mesothelium, most frequently in the pleura or peritoneum, but also in the pericardium or tunica vaginalis testis [15]. Also a similar case with malignant mesothelioma that infiltrated the pectoralis has recently been published [16].

 Although thoracoscopy is usually the routine procedure to obtain a pleural biopsy, an alternative method of biopsing the enlarged supraclavicular lymph node (Figure 1) was preferred to confirm the diagnosis of mesothelioma [13, 14]. The result of the biopsy was positive for malignant metastatic mesothelioma. A second biopsy was performed and the sample was sent to another pathology department, in order to exclude any possible error. The result of the second biopsy was also positive for metastatic malignant mesothelioma. Immunohistochemistry method was positive for the following staining profile: AE1/AE3, WT-1, and calretinin, D2-40 and negative for CEAp, TTF-1. The patient was stage IV according to the International Mesothelioma Group (IMIG) [17]. Unfortunately none of the pathology laboratories were able to define the subtype of the mesothelioma (epithelial, sarcomatoid, or mixed), which is a very important prognostic factor. The patient refused to undergo another diagnostic biopsy, since it was explained to him the extent of the disease and the cost effect of another biopsy, since it would not change his therapeutic treatment approach. The staging was done with CT scan of the thorax, abdomen, brain, and bone scintiscan. Due to the initial extent of the disease (supraclavicular lymph node), it was decided not to perform further diagnostic evaluation (e.g., PET scan).

The treatment of malignant pleural mesothelioma (MPM) has evolved in the past 20 years. Treatment selection was based on tumor stage and patient's overall medical condition. Two randomized trials have now established the combination of cisplatin and an antifolate pemetrexed or raltitrexed as the standard in the systemic therapy for mesothelioma [18, 19]. In a published study on those who actually received chemotherapy, immediate treatment was associated with a significantly longer time to symptomatic progression and a trend to improved overall survival: 66% at 1 year compared to the “delayed” patients (36%), whose quality of life was less well maintained. Whether the true improvement in median survival with chemotherapy will be in the observed magnitude of 3-4 months is doubtful. As is the case in advanced NSCLC, the estimated median improvement in a general mesothelioma population eligible for platinum-based chemotherapy will probably average 8–10 weeks [20]. Although, strictly speaking, head-to-head comparisons between cisplatin/pemetrexed, cisplatin/raltitrexed, MVP, or vinorelbine need to be conducted, it is foreseeable that, also in analogy with NSCLC, none of these regimens will prove to be superior and that criteria other than outcome will become decisive: toxicity, compliance, and ease of administration. We chose to treat the patient with carboplatin, gemcitabine and, pemetrexed as reported in other studies, due to the excellent performance status but also for psychological reasons to encourage the young patient. The multimodality treatment protocols have achieved a median survival of 19 to 46 months depending on the stage, histology, and completeness of the surgical resection [21–25]. In a certain subgroup of patients with epithelial histology, no lymph node involvement, and complete surgical resection, the results were even more favorable with occasional long-term survival [21]. During the evolution of the treatment of MPM, successful local control of the disease with acceptable morbidity and mortality has been achieved through extra pleural pneumonectomy (EPP) and adjuvant high-dose hemithoracic irradiation [22, 23, 26]. The feasibility of high-dose hemi thoracic irradiation following EPP was investigated in a phase II trial from Memorial Sloan Kettering Cancer center with local control rates over 90% [22, 26]. In a similar study from MD Anderson Cancer center, despite successful local control, distant recurrence, especially in the abdomen, was a major problem in long term [27]. The patient received six cycles of chemotherapy with initial response. A decrease in mediastinum and supraclavicular lymph nodes was observed. The inflammation and lymphatic obstruction on the right supraclavicular area was yielded. At his last followup, 2 months after his last chemotherapy session, the patient was referred to our department with extensive ascites, despite the stable disease on the thorax. It was decided to initiate chemotherapy with carboplatin AUC 5.5 and docetaxel 100 mg/m^2^, not only based on our center's experience and clinical practice, but also based on previous published trials [28, 29].

## 3. Discussion

The widespread use of asbestos during the 20th century has produced a legacy of ill-health, death, and contamination which will endure well into the current century. Mesothelioma is an incurable but rare disease with an incidence in industrialized countries ranging from 5 to 50/million/year [30]. This cancer is almost exclusively caused by exposure to asbestos. In 1960, Mossman and Gee reported the first conclusive association between asbestos exposure and pleural mesothelioma [31]. Pleural mesotheliomas, which represent the most common form, have a male to female ratio of about 5 to 1 [32]. Although the tumor mainly affects older men who in their youth were occupationally exposed to asbestos, malignant mesothelioma can also occur in children [33]. Epidemics of asbestos illness have been reported in industrialized countries for many years; now data are being collated, which document an escalation of these diseases in developing countries such as Brazil, Thailand, and Egypt. In Greece, 2% of the recognized occupational morbidity has been attributed to pleural mesothelioma (a single case of a sheet-metal worker in 2001 that was finally recognized as an occupational disease); however, there is a longstanding issue of underreporting and under-recognition of occupational diseases [34, 35]. However, we should be aware of the fact that MPM can also occur without exposure to asbestos fibres, but in our case the exposure of the patient to asbestos fibres was certain.

 Until 1995, Greece was amongst the world's top seven suppliers of asbestos, producing 100,000 tonnes of chrysotile every year with up to 300,000 tonnes a year of Greek and imported asbestos processed at various asbestos-cement factories. Asbestos-containing brakes and fireproofing materials were also produced in Greece. In 1993, the use of blue asbestos (crocidolite) was banned by law (article 1154/93); on December 31, 2004, Greece became the last of the 15 European Union (EU) Member States to ban the use of all forms of asbestos as per the EU Directive [34, 36]. There are six types of asbestos fibrous silicates (actinolite, asbestos grunerite, anthophyllite, chrysotile, crocidolite, and tremolite), all capable of causing mesothelioma; however, only three types have been used commercially. White asbestos (chrysotile), brown asbestos (amosite), and blue asbestos (crocidolite) have been used in the construction of school buildings. Amphiboles (crocidolite and amosite) are more potent than chrysotile alone [37]. Asbestos was most commonly used in schools as insulation and in building materials. It has been used in floor and ceiling tile, walls, acoustic insulation/attenuation, doors, blackboards, cement asbestos pipe, corrugated paper pipe wrap, decorative insulation, pipe and boiler insulation, and spray-applied fireproofing [38]. The amount of asbestos in these products varies widely from 1% to 100%, with pipe and boiler insulation containing more asbestos than any other building material. As the precise amount of asbestos in a product cannot often be determined on the basis of its label or by contacting the manufacturer, its positive identification requires analysis of samples by a qualified laboratory. 

 Asbestos-containing materials become hazardous when, due to accidental damage, vandalism, or deterioration over time, they release fibers that remain in the air for many hours. Heaps of asbestos wastes with breaks of cement were collected. Although the association between asbestos exposure and the development of malignant pleural mesothelioma (MPM) is well recognized, the relationship between asbestos contents by fiber type and the risk of development for MPM remains unclear. An association between and evaluation of asbestos and fiber-type concentration were made in a small study [39]. To elucidate the relation between the pulmonary asbestos-fiber contents by fiber type and development of MPM, further investigation is considered necessary. In our case, we did not try to match the tissue fibers of the biopsy to the fibers used for the school materials since we did not have access to the school debris. Finally, we do not have data about the incidence of mesothelioma in other individuals that were exposed to the building, due to the long time that has passed since our patient has lost contact with his teachers and classmates.

##  Consent

The authors acquired written informed consent from the patient to publish data and figures from his medical file.

##  Conflict of Interests

The authors declare no conflict of interest.
